# Biomimetic Dentin Repair: Amelogenin-Derived Peptide Guides Occlusion and Peritubular Mineralization of Human Teeth

**DOI:** 10.1021/acsbiomaterials.2c01039

**Published:** 2023-02-28

**Authors:** Deniz T. Yucesoy, Hanson Fong, John Hamann, Eric Hall, Sami Dogan, Mehmet Sarikaya

**Affiliations:** † Department of Materials Science and Engineering, University of Washington, Seattle, Washington 98195, United States; ‡ Department of Bioengineering, Izmir Institute of Technology, Urla, Izmir 35430, Turkey; § Department of Restorative Dentistry, University of Washington, Seattle, Washington 98195, United States

**Keywords:** remineralization, demineralization, occlusion, exposed dentin tubules, penetration

## Abstract

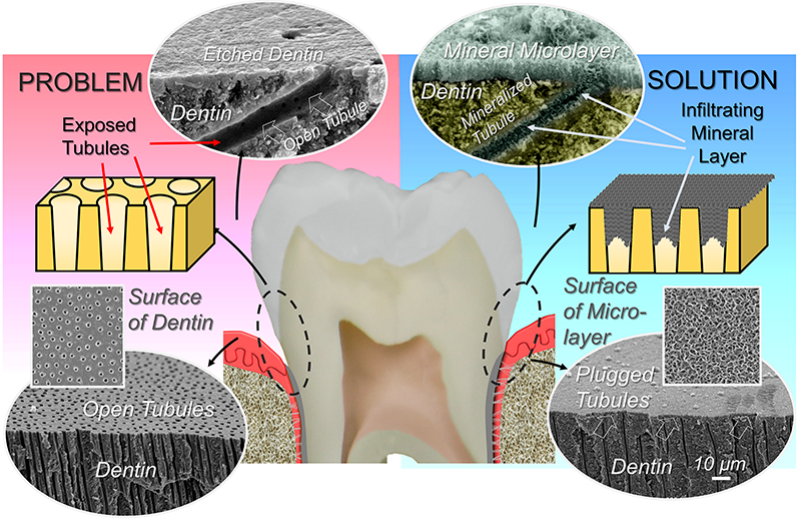

Exposure of dentin tubules due to loss of protective enamel (crown) and cementum (root) tissues as a result of erosion, mechanical wear, gingival recession, etc. has been the leading causes of dentin hypersensitivity. Despite being a widespread ailment, no permanent solution exists to address this oral condition. Current treatments are designed to alleviate the pain by either using desensitizers or blocking dentin tubules by deposition of minerals or solid precipitates, which often have short-lived effects. Reproducing an integrated mineral layer that occludes exposed dentin with concomitant peritubular mineralization is essential to reestablish the structural and mechanical integrity of the tooth with long-term durability. Here, we describe a biomimetic treatment that promotes dentin repair using a mineralization-directing peptide, sADP5, derived from amelogenin. The occlusion was achieved through a layer-by-layer peptide-guided remineralization process that forms an infiltrating mineral layer on dentin. The structure, composition, and nanomechanical properties of the remineralized dentin were analyzed by cross-sectional scanning electron microscopy imaging, energy dispersive X-ray spectroscopy, and nanomechanical testing. The elemental analysis provided calcium and phosphate compositions that are similar to those in hydroxyapatite. The measured average hardness and reduced elastic modulus values for the mineral layer were significantly higher than those of the demineralized and sound human dentin. The structural integration of the new mineral and underlying dentin was confirmed by thermal aging demonstrating no physical separation. These results suggest that a structurally robust and mechanically durable interface is formed between the interpenetrating mineral layer and underlying dentin that can withstand long-term mechanical and thermal stresses naturally experienced in the oral environment. The peptide-guided remineralization procedure described herein could provide a foundation for the development of highly effective oral care products leading to novel biomimetic treatments for a wide range of demineralization-related ailments and, in particular, offers a potent long-term solution for dentin hypersensitivity.

## Introduction

Dentin hypersensitivity (DH) is a prevalent condition ranging from 42% of the adult population^1^ to 72.5–98% of the periodontal patients.^2^ Exposure of tubular dentin due to the loss of protective mineralized tissues, e.g., enamel at the crown and cementum on the root of the tooth, is one of the leading causes of DH.^3−5^ While the loss of enamel can be due to abrasion, caries, bruxism, or abfraction,^5^ the gingival recession and subsequent loss of cementum often occur as a result of aggressive oral hygiene measures, aging, or periodontal diseases.^3^ Widely accepted hydrodynamic theory states that the fluid movement within the exposed dentin tubules in response to external stimuli can cause pressure fluctuations across dentin, stretching or compressing the nerves located at the pulp, which results in a pain response.^6^ DH often affects patients’ quality of life by changing dietary habits and oral hygiene measures to avoid pain stimulation and, thus, increases the risk of other dental problems, most notably, caries and periodontitis.^7,8^ Additionally, in recent years, frequent use of peroxide-based dental whitening products further exacerbated this serious dental problem.^9,10^

Despite being a prevalent dental condition with an upward trend of risk, there has not been a permanent solution to DH.^11,12^ The existing products (including the toothpastes) are designed to alleviate the pain by either (i) desensitizing the nerves by using ingredients such as KNO_3_^12,13^ or (ii) physically blocking dentin tubules to reduce the fluid flow by solid precipitates, e.g., oxalates, strontium salts, calcium fluoride, arginine with calcium carbonate, amorphous calcium phosphate, and bioglasses.^14−17^

Many of the products of the latter group were shown to reduce dentin permeability in 4–6 weeks of treatment.^14,17^ These effects, however, are often short-lived, leading to re-exposure of tubules and resumption of the DH symptoms.^18,19^ Although the chemical stability of the occlusion layer has been demonstrated to be markedly improved using the ionic additives, e.g., Sr^2+^ and F^–^, the limited peritubular mineralization capacity of these procedures has been a persistent shortcoming, making the newly formed surface layer susceptible to mechanical wear and thermal delamination.^20−22^ Among these, occlusion agents such as calcium carbonate and nanohydroxyapatite mostly result in deposition of large precipitates, which tend to agglomerate at the tubule entrance and, thus, prevent mineral infiltration.^11,23−25^ Similarly, uncontrolled and rapid epitaxial growth of minerals from ionic precursors, supplied in high concentrations, often blocks the dentin tubules at the surface before the peritubular mineralization is established.^26^ Tubules that remain exposed due to incomplete occlusion or the breaches in the integrity of the surface layer as a result of cyclic stresses could provide potential routes for bacterial infection, which can progress into pulp and lead to infection of the root canal system.^27,28^ Alternatively, stabilizing agents, such as casein phosphopeptides or cations for Ca^2+^ substitution, e.g., Mg^2+^, Sn^2+^, etc., have been demonstrated to inhibit spontaneous precipitation by slowing down the ion release. These approaches, however, work at the expense of mineral formation, necessitating significantly longer treatment time to facilitate sufficient mineralization that can effectively cover exposed tubules.^28−30^ Besides being unreasonably long for practical implementations, there are substantial disagreements among the experts with respect to remineralization potential of many of these approaches.^17,26,31,32^

As we offer in this work, controlling the dentin remineralization process with accelerated kinetics using short peptide domains could be an alternative solution to ensure sufficient peritubular mineralization before the tubule entrances are blocked with the newly forming mineral layer on the exposed dentin surface.^24,33,34^ Referred to as amelogenin-derived peptides (ADPs), these 15–25 amino acid long peptides have been demonstrated to facilitate the control of mineralization on human enamel and sound radicular (root) dentin.^24,35^ The objective of the current study is to develop a peptide-guided biomimetic remineralization model that could eventually be used as a guide to repair exposed human dentin. Here, we demonstrate the formation of an integrated mineral layer with a chemical content similar to the existing natural tooth mineral on exposed dentin that not only structurally integrates into the underlying dentin but also infiltrates into exposed tubules via peritubular mineralization. The integrated mineral–dentin interface and peritubular mineralization are two essential characteristics to reestablish the structural integrity of the tooth with permanent functional durability.^26^ The structural, chemical (microstructural and compositional integrity across the interface), and mechanical characteristics of the resulting mineral layer have been evaluated by imaging and elemental analysis using scanning electron microscopy, nanomechanical tests, and thermal cycling. These tests demonstrate the reestablishment of the structural–functional, chemical, and mechanical integrity of the damaged tooth by the new mineral layer toward achieving a long-term durability. By harnessing the highly efficacious remineralization potential of the mineralization directing peptide to restore exposed dentin lesions, the procedures outlined in this work have the potential to be implemented into biomimetic oral care treatments with a lasting outcome for a wide range of demineralization-related dental ailments including dentin hypersensitivity.

## Experimental Section

### Materials and Methods

#### Peptide Design and Characterization

The amelogenin-derived peptide (ADP5) used in this work was originally designed using a procedure that was developed for deriving peptides with retained key functions from natural proteins, e.g., amelogenin, the key protein in enamel formation.^35^ A combination of experimental (including directed evolution using phage display library, binding properties using quartz crystal microbalance, and biomineralization characteristics using X-ray diffraction/electron microscopy) and computational approaches (bioinformatics, similarity analysis, and de novo design) were utilized to design, rigorously refine, and quantitatively characterize the predicted functions of the amelogenin-derived peptides. In this work, to further improve the aqueous solubility of 25-amino acid long ADP5 peptide, an essential propensity for future clinical applications, the hydrophobic domains in the amino and carboxyl-end were eliminated via systemic mutations, while keeping the charged amino acids intact that facilitate the hydroxyapatite (HAp) binding and mineralization. The retained catalytic activity of the resulting 15-amino acid long peptide, dubbed as sADP5, i.e., shortened ADP5, was characterized through the calcium depletion assay^36^ described previously (short synopsis of the Peptide Design and Characterization and Peptide Synthesis and Purification procedures are provided in Sections S1 and S2). Briefly, 0.8 μM peptide solution was mixed with an equal volume of mineralization solution containing 48 mM CaCl_2_·2H_2_O and 28.8 mM β-glycerophosphate (β-GP) in 25 mM Tris-HCl buffer (pH 7.4) to have a final concentration of 0.4 μM peptide, 24 mM CaCl_2_·H_2_O, and 14.4 mM β-glycerophosphate (β-GP). The mineralization reaction was started by adding 0.10 U/μL bacterial alkaline phosphatase (AP, Invitrogen, USA) into 200 μL of reaction mixture. As the negative control, an equal volume of 25 mM Tris-HCl buffer (pH 7.4) was added into the mineralization solution. Recombinant amelogenin (rm180), original ADP5, and phage-display selected HABP1 with slow kinetics were used as internal controls. Ten μL of the reaction solution was collected at 15, 30, 60, and 90 min. The reaction is stopped by quenching the AP activity by heating the solution to 90 °C and then rapidly cooling down to −20 °C. The mineral phase was removed by centrifugation, and the unreacted ionic calcium in the supernatant was measured using a QuantiChrome Calcium Assay Kit (Bioassays, USA).

#### Sample Preparation and Remineralization Procedure

Extracted human molar teeth (excluding third molars) with no visible defects or restorations were collected from University of Washington School of Dentistry Clinics in 10% (v/v) bleach solution under informed consent by Human Subject Division (University of Washington) and the approval for nonidentifiable specimen use. Bleach could react with the proteins leading to not only deproteination via nonspecific degradation but also altering the hierarchical structure of the collagen. To mitigate the effect of bleach, cervical dentin was extracted from the central region of the human tooth, which is covered with a thick layer of enamel and mantle dentin, to ensure that the dentin specimen used in this study was not in direct contact with the bleach solution. The exposed human dentin specimens were prepared according to the dentin disc model,^37,38^ which was developed for the evaluation of restorative treatments for dentin hypersensitivity. Briefly, 25 dentin discs with a thickness of ∼2 mm were cut from midcoronal dentin using a diamond blade. Coronal surface was then polished to the 0.1 μm finish. The specimens were ultrasonicated for 2 min and then etched for 30 s with 10% (w/v) citric acid solution to remove the smear layer. Prior to the remineralization treatment, the specimens were prewetted with 20 mM Tris buffer solution, pH 7.4 (TBS). Next, the dentin discs were placed into 0.8 mM sADP5 peptide solution^24^ and incubated for 10 min at 37 °C. Samples were then transferred into 20 mM TBS containing 3.22 mM Ca^2+^/1.92 mM PO_4_^3–^ for 1 h at 37 °C (see Section S3).

#### Characterization by SEM and EDXS Analysis

Prior to imaging, samples were fractured to expose the cross-section of the mineral layer and coated with 5 nm of Au–Pd for establishing electron conductivity. Scanning electron microscopy (SEM; JSM 7000F; JEOL-USA Co., Peabody, MA, USA) was used for surface topography and cross-sectional structure characterization using the secondary electron imaging mode. The SEM was operated at 8 kV accelerating voltage to reduce possible e-beam radiation damage. Elemental composition analysis was performed using an onboard energy dispersive X-ray spectrometer (EDXS; EDAX Inc., Mahwah, NJ, USA). It is noted that a freshly fractured geological apatite (originated from Sapo Mine, Goiabera, Minas Gerais, Brazil) was utilized as a reference material for elemental analysis (see Section S4 and Figure S2).

#### Nanomechanical Properties Characterization

The nanomechanical properties of the demineralized and remineralized dentin samples were characterized using a procedure described previously.^24,39^ In short, nanomechanical measurements were made using a Triboindentor nanoindentation system (Hysitron Inc., Minneapolis, MN, USA) in air with controlled humidity. To ensure volume dependent measurements, maximum indentation depth was kept at 100 ± 20 nm. All reported hardness, *H*, and reduced elastic modulus, *E*_r_, values were averaged over 20 measurements (a synopsis of the Nanoindentation Testing procedure is provided in Section S5 and Figure S3)

#### Thermal Aging Assay

The thermal aging of the remineralized dentin specimen was carried out by a thermal cycling assay that was adapted from the ISO/TS 11405 dental materials testing procedure.^40^ Briefly, the remineralized dentin discs were soaked in 10 mL of artificial saliva (containing 130 mM KCl, 20 mM HEPES, 1.5 mM CaCl_2_, 0.9 mM KH_2_PO_4_, and 1 mM NaCl; pH 7.0) and cycled for 200 and 2,500 rounds through two temperature extremes, 5 and 55 °C, with 30 s of dwelling time at each temperature. Samples were then fractured to expose the cross-section of the dentin–mineral layer interface, and structural and nanomechanical characterizations were performed using SEM and nanoindentation as described above.

#### Statistical Analysis

Quantitative data were presented as mean ± standard error from independent experiments (*n* = 5) using a Microsoft Excel Worksheet. The repeated measures analysis of variance (RM-ANOVA) test was performed to evaluate the calcium consumption profiles using IBM SPSS Statistics Version 25 (IBM SPSS, Chicago, IL). The level of significance was set to α ≤ 0.05. Tukey and Dunnett’s T3 posthoc tests were utilized to identify statistical significance between the individual test groups.

## Results

**Figure 1:**
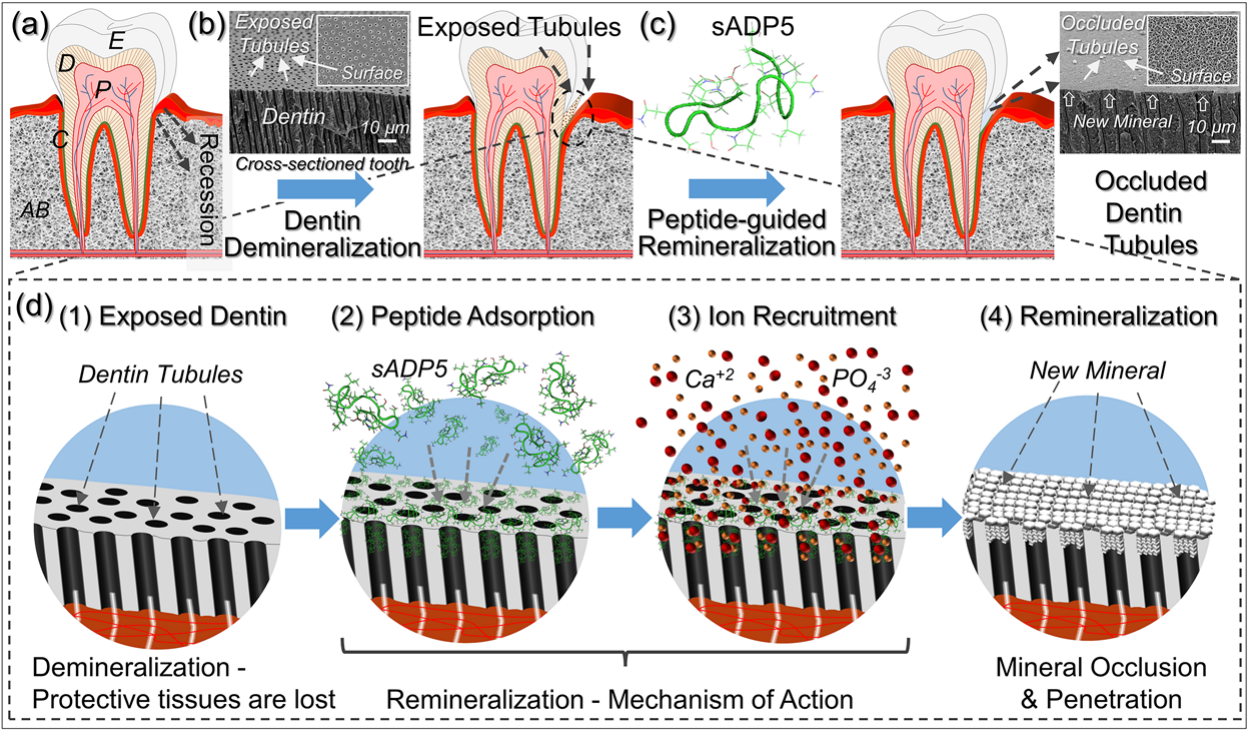
Peptide-guided biomimetic remineralization of exposed human dentin. (a) Schematic cross-section of a second molar displaying all five dental tissues. (b) SEM image of the exposed dentin surface decorated with open tubules as a result of recession at the gingival margin shown by arrows. (c) Peptide-guided remineralization covers the dentin surface and occludes the tubules, as shown in the inset (cross-section and face-on SEM images). (d) Mechanism of action in peptide-guided remineralization: (1) Dentin tubules are exposed to the oral environment as a result of demineralization of protective enamel or cementum tissues. (2) During the biomimetic restoration, mineralizing peptide binds to the exposed dentin surface. (3) Recruits Ca^2+^ and PO_4_^3–^ ions. (4) Guides growth of the hydroxyapatite mineral layer. The newly formed mineral occludes and penetrates into dentin tubules, thereby providing a well-integrated and durable mineral layer on dentin (AB: alveolar bone; C: cementum; D: dentin; E: enamel; P: pulp; sADP5: shortened amelogenin-derived peptide 5).

The key requisite for reestablishing integrity of the tooth that is worn out with exposed dentin is to form a structurally and functionally stable mineral layer on the surface that also penetrates into the dentin tubules. This is necessary to achieve a long-term durability of the newly formed mineral layer on teeth. In this work, restoration of the exposed human dentin was accomplished using a 15-AA long sADP5 peptide that guides calcium-phosphate remineralization with accelerated kinetics. The resultant structural characteristics were analyzed by scanning electron microscopy imaging using cross-sectioned samples that reveal the extent of the surface mineral layer and its penetration into the tubules. The peptide-guided remineralization process which takes place both on the surface of dentin and the peritubular space is schematically depicted in Figure 1. The outcome of the stability of the mineral formation and its integration through the biomimetic restoration process was also shown by thermal cycling, which simulates the dynamic conditions in the oral environment.

### Design and Catalytic Characterization of the sADP5

**Figure 2:**
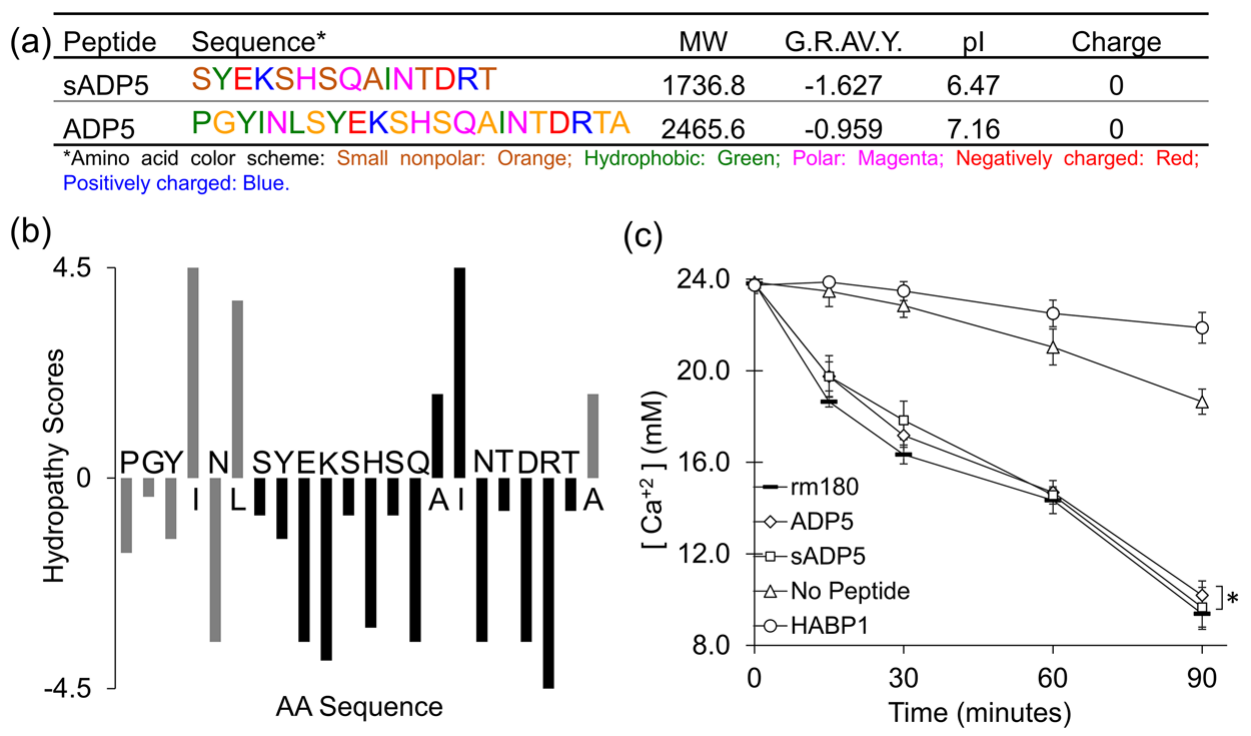
(a) Amino acid sequence and physical characteristics of sADP5 vs ADP5. (b) Hydropathy plots of ADP5 and sADP5 sequences based on Kyte-Doolittle scoring.^42^ (c) Calcium consumption profiles in the presence of recombinant amelogenin (rm180), combinatorially selected HABP1, and protein derived sADP5 and ADP5 peptides (*: no statistically significant difference).

The utility of 25 AA long ADP5 peptide in controlling the calcium phosphate mineralization on sound root dentin was reported previously.^35^ Using a postselection peptide engineering approach, a series of systematic mutations on the original sequence was carried out to increase the aqueous solubility of the peptide while retaining its catalytic activity in controlling the calcium phosphate mineralization both in aqueous solutions and the tissue surface. As a result, the original length of the ADP5 was shortened by eliminating the hydrophobic domains in the amino- and carboxyl-end of the ADP5, while keeping the charged amino acids intact, which initiate mineralization. The color-coded amino acid sequence of the modified peptide (according to Lesk et al.^41^) along with its hydropathy distribution based on Kyte-Doolittle^42^ scoring and selected biochemical properties are provided in Figure 2a,b.

The retained biomineralization activity of the resulting 15-amino acid long sADP5 peptide (shortened ADP5) was characterized using an alkaline phosphatase (AP)-based mineralization model,^35,36^ which mimics the biological matrix vesicle mediated mineralization process. As shown in Figure 2c, the sADP5 peptide exerted a mineralization activity similar to that of recombinant amelogenin (rm180) and the original ADP5.^35,36^ Compared to the no-peptide case, in the presence of sADP5, the mineralization reaction rate was increased about 2.5 times, leading to consumption of more than half of the available free calcium in 90 min at 37 °C. As internal control, the calcium consumption profile of another peptide, namely, HABP1, was also monitored, which was originally selected through directed evolution and is known to bind to hydroxyapatite mineral with high affinity.^36^ It should be noted here that the strong binding to a mineral is not necessarily coupled with the mineralization characteristics of a given solid-binding peptide as demonstrated with this calcium consumption test. In fact, the ion-consumption rate of this high affinity peptide was even slower than the no-peptide case. The three important steps of the peptide-guided biomineralization process, namely, mineral binding, formation, and morphogenesis, probably have more sophisticated foundational mechanisms and should be a subject of future explorations.

### Occlusion of Dentin Tubules through Peptide-Guided Treatment

Repair of enamel defects, such as white spot lesions and surface caries, would involve formation of a new layer that strongly binds to the surface of the hard tissue that is primarily mineralized.^43,44^ In the case of dentin, which is partially mineralized with large organic content, the surface mineralization is more challenging requiring the formation of mineral on a partially demineralized collagen network. It is also desirable for the newly forming mineral to penetrate into the dentin tubules to form an interdigitated interface, resembling the interlocking structure at the dentin–enamel junction.^43,45^

**Figure 3:**
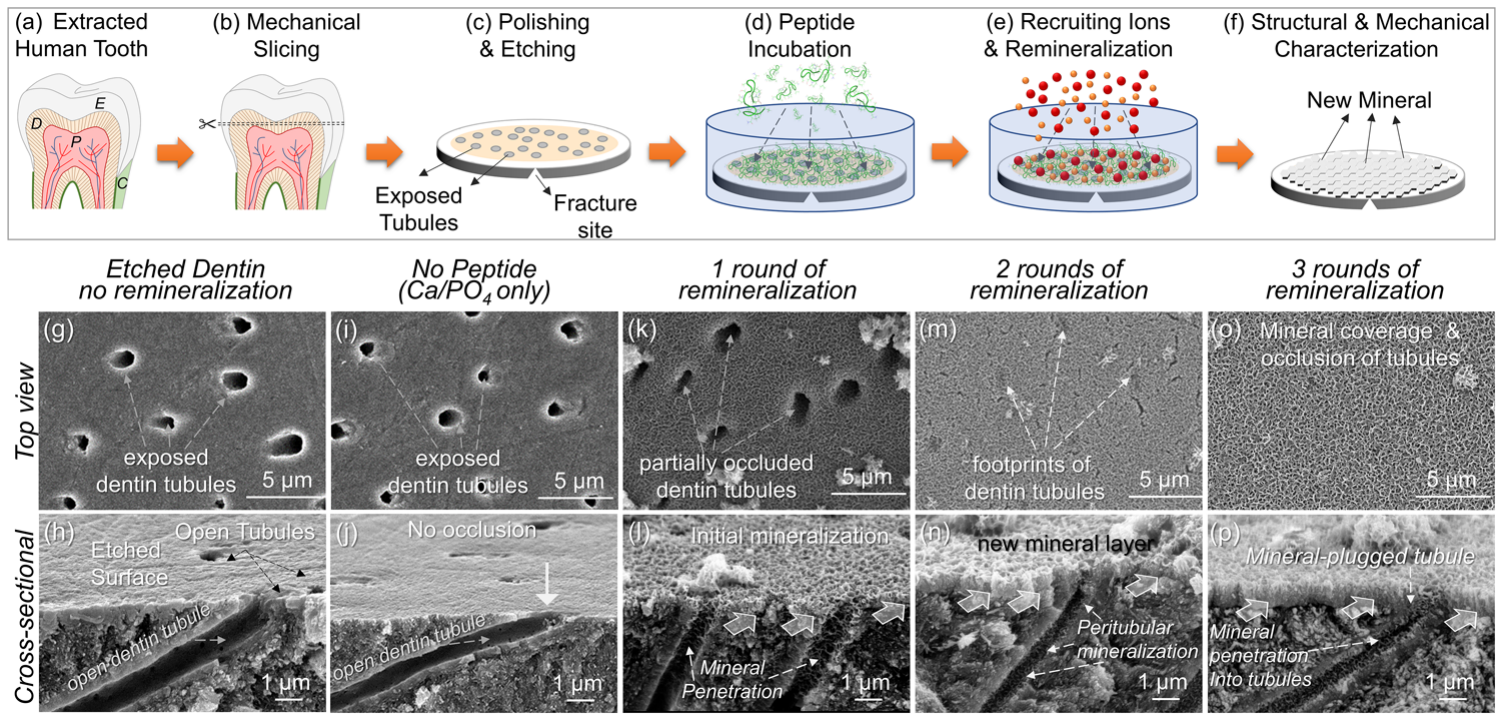
Remineralization cycle in vitro: (a) The extracted teeth samples were (b) mechanically sliced in the mesio-distal plane, (c) followed by mechanical polishing and etching (see Materials and Methods). The samples were (d) placed in an aqueous solution with dissolved peptides and then (e) transferred into a calcium and phosphate containing solution for (f) remineralization to take place. The newly formed mineral layer on the surface of the tooth was imaged in face-on and edge-on views (secondary electron images recorded by SEM). (g, h) Demineralized and chemically etched dentin. (i, j) Control specimen treated with calcium and phosphate solution in the absence of peptide. (k, l) Sample after a single round of mineralization, where a new mineral layer starts to form on the surface but tubules are still visible. (m, n) After two rounds of mineralization, where the newly formed mineral layer grows thicker on the surface (arrows) and footprints of exposed tubules are barely visible. (o, p) The sample with three rounds of mineralization, where newly formed mineral layer reached ∼2 mm thickness; dentin tubules are occluded completely, and the mineral layer penetrated the tubules.

Dentin discs obtained from extracted from human teeth were utilized to demonstrate the efficacy of peptide-guided remineralization treatment. The procedure includes cross-sectioning the tooth sample with open-ended dentin tubules (Figure 3a–c), followed by peptide exposure in an aqueous solution (Figure 3d) and finally placing the specimen into remineralization solution (Figure 3e,f). Formation of a new mineral layer on the exposed dentin was accomplished by three rounds of peptide guided remineralization treatment. The face-on and edge-on view of the cross-sectioned teeth samples show the tubules extending to the specimen surface, displaying a typical exposed dentin topography with open tubules (Figure 3g,h). The average dentin tubule diameter was measured as 2.0 ± 0.5 μm. Incubation of the specimen inside the remineralization solution containing ionic calcium and phosphate in the absence of peptide (control group) resulted in uneven deposition of unstable tiny crystals on the demineralized dentin surface, which were washed off from the tissue surface by a deionized water rinse (Figure 3i,j). On the other hand, a single round of peptide-guided remineralization treatment resulted in a 0.8 ± 0.3 μm thick continuous mineral layer on the dentin surface (Figure 3k,l). The SEM image obtained from the cross-sectioned mineralized dentin clearly demonstrates that a newly formed mineral covering the dentin surface also extends into the dentin tubules. At this point, the occlusion of dentin tubules was not completed, although the mineral formation within the tubule entrance reduced the average diameter of the dentin tubules by 0.75 ± 0.5 μm (Figure 3l).

The second round of mineralization treatment resulted in the addition of more mineral on top of the first layer. Through the layer-by-layer mineralization process, the mineral thickness reached 1.1 ± 0.4 μm. Upon this treatment, the footprints of exposed tubules were barely visible and tubule occlusion on the surface was close to being completed (Figure 3m,n). After the third round of remineralization treatment, tubule occlusion was completed on the surface while the mineral penetration into the tubule advanced deeper, forming a tapered geometry (Figure 3o,p). Upon this treatment, the average size of the tubules became 0.7 ± 0.2 μm at 10 μm depth from the surface (Figure 3p). The average thickness of the mineral layer facilitating the complete occlusion of the tubules on the exposed dentin was recorded as 2.1 ± 0.4 μm.

### Elemental Composition of Newly Formed Mineral Layer

**Figure 4:**
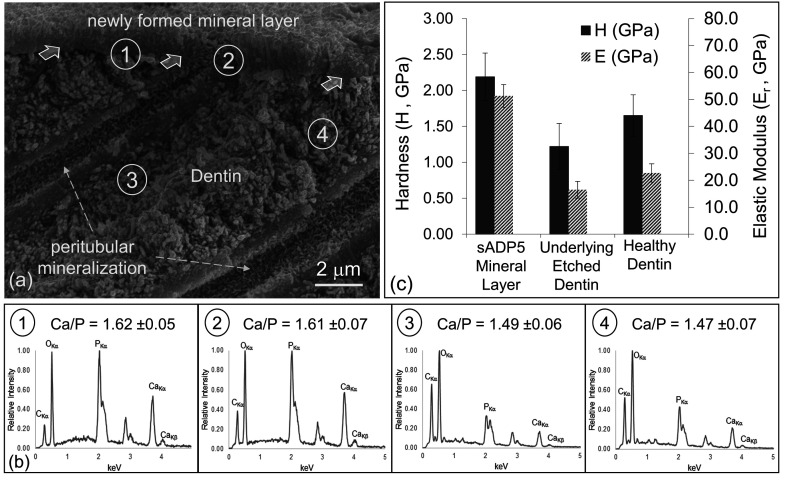
Compositional and mechanical properties analyses of the newly formed mineral on dentin. (a) The cross-section teeth specimens (imaged by SEM in (a)) were used for the elemental composition analysis that was carried out by acquiring EDXS spectra from four different regions (b) 1 through 4. The shown Ca/P ratios were determined using the Kα peaks of the Ca and P from the spectra. (c) Mechanical properties were determined using nanoindentation at spatial positions using the cross-sectioned samples exposing the sound dentin, etched tooth, and newly formed mineral. Both the hardness and elastic modulus of the newly formed mineral display statistically higher values than sound dentin, while the etched dentin surface, mimicking demineralization, shows lower values, possibly due to mineral loss.

The identity of the newly formed mineral on the dentin surface was ensured via elemental compositional analysis for Ca and P using energy dispersive X-ray spectroscopy and compared with the Ca/P ratio of the stoichiometric hydroxyapatite (Figure 4a,b). For this analysis, dentin specimens treated with 3 rounds of remineralization were utilized as this treatment produces the thickest mineral layer. For SEM and EDXS analysis, samples were prepared in cross-sectional orientation displaying all the regions involved, including sound and demineralized dentin, newly formed mineral layer, and peritubular mineral on the inner surface of the dentin tubules.

The spectra 1 and 2, shown in Figure 4b1,b2, were acquired from the mineral layer on dentin and at the tubule end. These spectra display prominent Ca Kα and P Kα peaks with a Ca/P ratio of 1.62 ± 0.05 and 1.61 ± 0.07, respectively. This ratio is close to the ideal ionic ratio of 1.66 in geological apatite and HAp composition (see Section S4). The spectra 3 and 4 were acquired from the dentin at spatial positions 1 and 5 μm below the interface (Figure 4b3,b4). These spectra reveal characteristic Ca Kα and P Kα X-ray peaks with an Ca/P intensity ratio of 1.49 ± 0.06 and 1.47 ± 0.07, respectively. These values are smaller than the ideal Ca/P ratio of the natural HAp mineral. The imbalance in the Ca/P ratio is an indication of the effect of surface preparation of the tooth specimens that was intended to mimic natural demineralization. It appears that, during the process, calcium preferentially leaches out down to the depth extending ∼10 μm below the sample’s original surface.

### Nanomechanical Properties of the Remineralized Layer on Dentin

Toward achieving the desired performance of the remineralized dentin and ensuring its long-term durability, their continuity of the structural and functional characteristics across the dentin–mineral layer interface need to be ensured. As discussed above, structural interpenetration of the newly formed mineral into the dentin tissue is achieved through layer-by-layer treatment using three rounds of peptide-guided mineralization. Because of the confined geometries of the structures involved, the nanoindentation testing was performed in cross-section geometry to determine the mechanical properties at local regions, including a newly formed mineral layer on the surface and the underlying dentin. Nanomechanical tests were also carried out on the freshly prepared samples that mimicked the demineralization process (as discussed in Figure 4). Force-deflection curves obtained during the nanoindentation test provide both the hardness, *H*, and the reduced elastic modulus, *E*_r_, of all three the regions of interest (see the Supporting Information for details of nanomechanical tests). The *H* and *E*_r_ values of the mineral layer were found as 2.19 ± 0.33 and 51.3 ± 4.2 GPa, respectively (Figure 4c). These values are, respectively, higher than those of the sound dentin, i.e., 1.22 ± 0.32 and 16.5 ± 3.1 GPa, determined from the interior of the sample. Not quite resembling the values of sound enamel, the fact that the newly formed mineral layer is harder and stiffer than those of the dentin is a desirable combination of mechanical properties suggesting that the newly formed mineral on the dentin surface mimics interlocking tissue junctions, e.g., dentin–enamel junction, both structurally and functionally. For the simulated natural demineralization process in the oral cavity, dentin samples were etched to partially leach out the surface mineral. The nanomechanical tests conducted on the surface of the freshly prepared tooth samples prepared for remineralization (see Figure 4a) produced values of hardness and elastic modulus, i.e., 1.65 ± 0.29 and 22.7 ± 3.4 GPa, respectively, that are significantly lower than those of the sound dentin. These inferior mechanical properties of the surface layer of the dentin are, therefore, reflective of demineralization treatment and consistent with the compositional analysis that also showed preferential calcium loss from regions of the samples close to the surface.

### Thermal Durability of the Newly Formed Mineral Layer on Dentin

**Figure 5:**
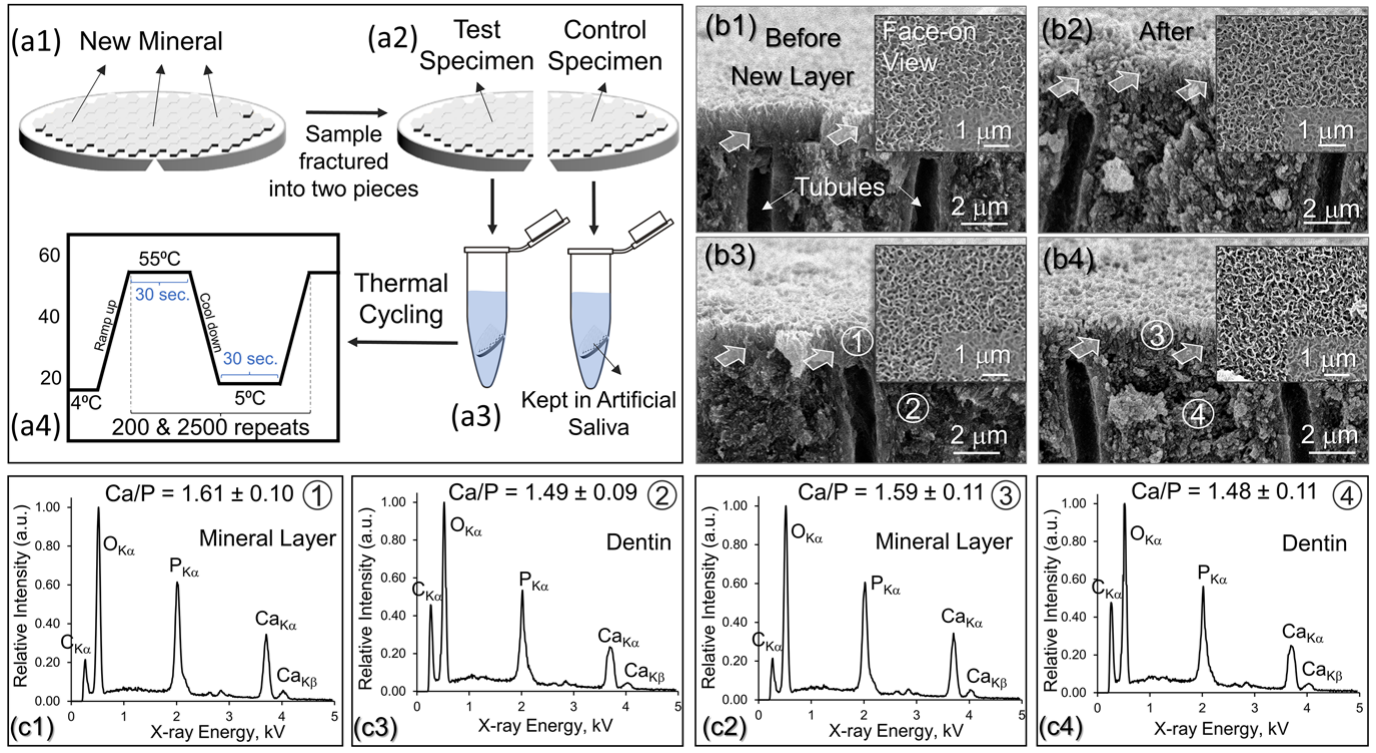
Schematic representation of thermal testing procedure. (a1) Remineralized dentin was (a2) fractured into two halves and placed into artificial saliva solution. (a3) While the control specimen was kept at 4 °C during the procedure, (a4) the test specimen was placed in a thermal cycler and subjected to thermal agitation between 5 and 55 °C for 200 and 2500 cycles to simulate 6 days and 3 months of thermal aging, respectively. (b) Structural and elemental compositional characterization after thermal aging treatment: Images show the edge-on view, and insets are the face-on view of the newly formed mineral on previously exposed dentin covered with a new mineral, all recorded by SEM. Micrographs show remineralized dentin (b1) before and (b2) after 200 cycles of thermal aging. (b3, b4) SEM micrographs showing remineralized dentin before and after, respectively, after 2500 cycles of thermal aging. Elemental composition analysis on newly formed mineral layer (c1) and underlying etched dentin (c3). Spectra collected from the locations highlighted in (b3) as 1 and 2. Elemental composition analysis on newly formed mineral layer (c2) and underlying etched dentin (c4) after cycles of thermal aging. Spectra collected from the locations highlighted in (b4) as 3 and 4.

The structure–property–chemistry relationships established above suggest that the remineralized microlayer on dentin could be effective; it is also imperative to ensure the mechanical durability over time. In this work, we also assessed the mechanical and structural durability of the newly formed mineralized layer through a thermal cycling assay (Figure 5). The test was designed to simulate the daily thermal changes in the oral cavity, e.g., consumption of hot and cold beverages or food, by exposing the remineralized specimen between the two extremes of 5 and 55 °C (Figure 5a1–a4; more details of the test; see Section S6). Three rounds of remineralization treatment resulted in a 2.0 ± 0.3 μm thick continuous mineral layer occluding dentin tubules (Figure 5b1,b3). The thermal tests were conducted with 200 and 2,500 rounds of thermal cycling, which resulted in no significant change in the overall thickness of the mineral on the dentin surface (Figure 5b2,b4, respectively). The continuity of the mineral layer was also preserved and, more significantly, without delamination during the thermal cycling treatment (Figure 5b2,b4). The significance of this result is that no separation was observed at the mineral–tissue interface, which is a strong indication that the newly mineralized microlayer has structurally integrated into the underlying dentin to form a continuous interlocking interface leading to long-term durability.

Elemental composition analysis of the newly formed mineral layer and underlying demineralized dentin before and after 2,500 rounds of thermal agitation was also performed using the EDXS analysis (Figure 5c1,c4). The spectra were collected from the local positions on the newly formed mineral and the sound dentin. The spectra acquired from the mineral layer before and after thermal cycling (Figure 5c1,c3, respectively) revealed prominent Ca Kα and P Kα characteristic elemental X-ray peaks with a ratio of their intensities being 1.61 ± 0.10 and 1.59 ± 0.11, respectively.

The similarity of the elemental ratios demonstrates that there is no significant difference between the chemistries of the mineral layer before and after the thermal cycling process. Similarly, the spectra acquired from the underlying demineralized dentin before and after thermal cycling (Figure 5c2–c4) showed calcium-to-phosphorus ratios of 1.49 ± 0.09 and 1.48 ± 0.11. These values are consistent with the elemental composition of demineralized dentin described earlier (Figure 4). These calcium and phosphorus compositions after the thermal cycling processes confirm that there is persistent chemical stability of the mineral across the interface during the long-term thermal cycling process.

## Discussion

In this study, we demonstrated a cell-free, biomimetic restoration of demineralized human dentin with exposed tubules using the peptide-guided remineralization process. The premise of this approach is to guide mineralization on the worn-out tooth surface similar to the extracellular matrix proteins, e.g., amelogenin, directing mineral formation in odontogenesis.^44,46,47^ The focus of the present study, therefore, was to establish a protocol that not only forms a mineral microlayer on the surface of the exposed dentin but also physically plugs the dentin tubules via peritubular mineralization, which has been a long-standing challenge in dentin repair.^48^ The common limitation of the current treatments is the uncontrolled precipitation or accumulation of organic deposits or inorganics minerals on the dentin surface via secondary mineralization resulting in highly limited structural and functional integration into the underlying dental tissue.^49^ The goal has, therefore, been to utilize biological systems, e.g., cells, or biomimetic routes, e.g., proteins or peptides, to direct calcium-phosphate mineralization to the tooth surface. Called primary mineralization, the new microlayer formed using these strategies is expected to have structural and mechanical characteristics that enable establishment of a coherent, interlocking interface that is similar to dentin–enamel and dentin–cementum junctions.^43,45,50^

As we demonstrated herein, the etched midcoronal dentin discs from human subjects, mimicking demineralized exposed dentin, i.e., prevalent cause of dentin hypersensitivity, were subjected to repeated rounds of remineralization treatment. It is significant to note that the objective of utilizing midcoronal dentin is to obtain high density dentin tubules aligning perpendicularly to the surface. The resulting mineral layer was characterized structurally by SEM and chemically by EDXS for the interface and elemental composition analyses, respectively. The face-on and edge-on views of demineralized tooth samples displayed an exposed dentin structure with open dentin tubules on the surface (Figure 3g,h). The repeated rounds of remineralization treatment facilitated further growth of minerals on dentin (Figure 3k–p) through layer-by-layer mineralization mechanisms. The occlusion of dentin tubules requires the binding of the peptide to the surface of dentin and diffusion into the tubules recruiting the ions to eventually promote mineralization on the walls of the tubules. The tapered mineralized layer within the tubules demonstrates the extent of peritubular mineralization (Figure 3l,n,p). Complete occlusion of tubules was observed after the third round of treatment with a final thickness of 2.1 ± 0.4 μm on the surface with concomitant thickening of the penetrated mineral within the tubules. The conical shape of the tubules after three rounds of mineralization suggests that mineral growth starts as the tubule entrance (surface) progresses bidirectionally forming both on the surface and inside the tubules. At the time when the occlusion was completed, the average size of the tubules at the first 10 μm region from the surface was reduced to about 1/3 of its original diameter with the penetration of a newly formed mineral layer into the dentin tubules (Figure 3p). The elemental analysis of the new mineral layer provided a calcium-to-phosphorus ratio that is similar to that of natural hydroxyapatite indicating that the newly formed mineral has physical and chemical compatibility with the underlying dentin (Figure 4).

The main cause of the failure of dental restorations in general, occlusion agents in particular, is due to the lack of physical, chemical, and structural compatibility between the restorative material and the underlying tissue.^5,24^ The continuous occlusal forces as well as the thermal changes in the oral environment accelerate the mechanical wear, induce cracks, and cause formation of gaps with eventual delamination at the interface leading to failure of the treatment. The delamination would also provide an environment in which cariogenic bacteria can spread and progress into the dentinal tubules resulting in secondary caries formation as well as the infection of the root canal system.^27,51^ The degree of penetration of the newly formed mineral inside the dentin tubules and the establishment of a well-integrated interface are critical attributes that premise the long-term durability of the peptide-guided mineralization treatment.^26^ To ensure this, the mechanical compatibility and thermal durability of the remineralized layer were investigated using nanoindentation and thermal aging. The average hardness and elastic modulus values for the mineral layer were found to be significantly higher than those of the demineralized and sound human dentin but lower than the healthy enamel.^24,52^ The thermal cycling assay carried out to assess the thermal durability of the mineralized layer in the artificial saliva showed no significant change in the overall thickness, morphology, or composition of the newly formed mineral on the dentin surface. More importantly, the continuity of the mineral layer was preserved during thermal aging (Figure 5b1–b4). It should be emphasized here that, in an otherwise weak mineral layer–tissue interface, the newly formed microlayer would separate readily from the underlying dentin due to the stresses propagated at the mineral microlayer–tissue junction due to the differences between their structural, chemical, and thermal properties. The delamination, therefore, would be strong evidence of the interface fragility, which are common limitations of the glass, precipitate, or organic matter deposition-based treatments.^43,53^ As we have demonstrated in this work, the mineral layer–dentin interface remained integrated with no marginal separation, a strong indication that it is continuous, establishing a structurally robust and mechanically durable transition zone capable of withstanding long-term thermal stresses encountered in the oral environment. In natural tooth, the hard enamel tissue, i.e., crown of the tooth, or cementum, on the root, forms a structurally and functionally integrated transition zone with the underlying dentin, which is softer because of the high organic content, forming an interlocked dentin–enamel junction (DEJ) or dentin–cementum junction (DCJ).^43,45,50^ In this scenario, while the enamel forms a physicochemical buffer zone, withstanding everyday mechanical wear and tear, the underlying dentin provides toughness absorbing mastication stresses transferred through the strong interface, for the integrity of the overall tooth.^43^ Similar to the biological DEJ, as we demonstrate here, the hard and protective newly formed mineral microlayer (mimicking enamel) on the surface establishes a transition zone with the underlying dentin effectively creating, what may be called, a biomimetic mineral–dentin junction (MDJ) and, thereby, ensuring a long-term viability of the tooth.

**Figure 6:**
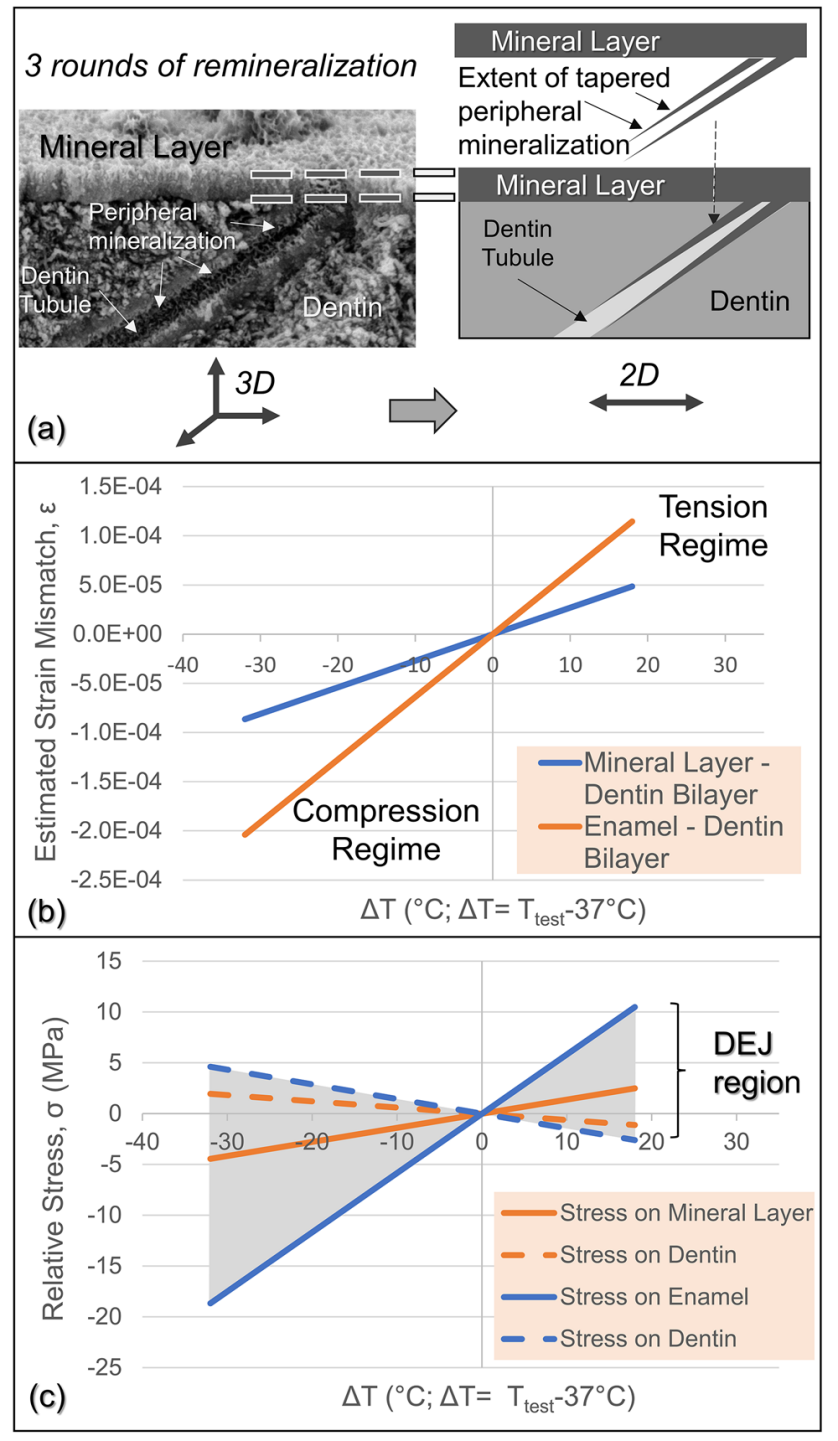
Estimated strain–stress analysis between sound enamel, dentin, and newly formed mineralized layers at mineral layer–dentin and dentin–enamel interfaces. (a) Simplified thin film approach is applied to estimate the thermal mismatch strain model, where the newly formed mineral layer is assumed to be contiguous and thin, ignoring the tubules in dentin that extend to the top. (b) The strain mismatch between the mineral layer and dentin (orange line) vs that between enamel and dentin (blue line). (c) The relative stress between mineralized layer and dentin in a dentin–mineral bilayer (orange line) and that between enamel and dentin in a dentin–enamel bilayer (blue line). Shaded region between dotted and solid blue lines represents the varying values within the DEJ. Compressive and tensile stresses are represented by negative and positive values, respectively.

We also emphasize here that the difference in the coefficient of thermal expansion (CTE) between the dental tissue and the restorative material is critical for the durability of the restoration.^49^ Continuous expansions and contractions at the tooth–restoration interface develops stresses due to the difference in the CTEs of the dentin tissue and the restorative material.^43,53^ If these stresses are large enough, then they may lead to propagation of cracks at the restoration margin leading to a premature failure of the interface. Using a thin film approximation (Figure 6a), the estimated strain and stress analyses were carried out among sound enamel, dentin, and newly formed mineralized layers at mineral layer–dentin and dentin–enamel interfaces (a short synopsis of the analysis is provided in Section S7). The strain and stress values between (i) enamel and dentin in a dentin–enamel bilayer and (ii) mineralized layer and dentin in a dentin–mineral bilayer are calculated from the differences of thermal expansion coefficients over the temperature range of thermal cycling (5–55 °C). The nanomechanical properties of the new mineral layer were found to be in line between those of the sound enamel and the sound dentin (Figure 6b,c). This highly critical result, therefore, ensures that a structurally well-integrated interface transition region was constructed between dentin and newly formed mineral.^43,54^ In addition, the estimated thermal mismatch strain between the dentin and mineralized layer is less than that of enamel over the same range of temperatures. These provide a projected range of thermal strain of 0.02% in tension to 0.04% in compression for the mineralized layer reflecting in the range of stresses between 2 MPa in tension to 3 MPa in compression. Strain delocalization plays a significant role in the reduction of thermal mismatch strain across the mineralized interface, which contributes to the structural integrity observed during the thermal aging process. The fact that the thermal stresses were not critically high to cause any delamination is a strong manifestation that the newly constructed mineral guided by sADP5 builds a structurally robust interface with the underlying dentin.

## Conclusions

In summary, this work demonstrates biomimetic restoration of artificially demineralized human dentin through peptide-guided remineralization. While in the natural process, proteins, e.g., amelogenin, play the key role in mineral formation, in the biomimetic process, primary dental remineralization is established by an amelogenin-derived peptide, sADP5. The occlusion of exposed dentin tubules is herein realized through a layer-by-layer mineral formation on the surface of the dentin that penetrates into the tubules covering the tubule walls thereby restoring the tooth’s natural protection. The procedures developed herein may provide a guidance toward addressing the challenge of treating dentin lesions and hypersensitivity. Further research concerning the permeability and chemical stability of the mineral layer is necessary to achieve an effective, easy-to-apply potent hypersensitivity treatment. Future studies would likely include implementing the peptide-guided remineralization approach under in vivo conditions (e.g., rat model) by utilizing a clinically applicable peptide delivery system, e.g., mineralizing gel or paste, and carrying out additional assays (pH cycling, dye penetration, etc.) to the mineralized layer to characterize its molecular adherence, sealing efficacy, and the chemical durability of the mineral–tooth interface toward more practical dental restorative materials and treatment procedures.

## Supporting Information Available

Detailed description of the (i) peptide design and characterization, (ii) peptide synthesis and purification, (iii) sample preparation and remineralization procedure, (iv) EDXS analysis of geological apatite, (v) nanomechanical characterization via nanoindentation testing, (vi) mineral durability characterization via thermal cycling procedures, and (vii) thermal strain and stress mismatch analysis (PDF)
