# Supplementary material for: Biomimetic Dentin Repair: Amelogenin-Derived Peptide Guides Occlusion and Peritubular Mineralization of Human Teeth
Source: ACS Biomater Sci Eng. 2023 Feb 28;9(3):1486–95. doi: 10.1021/acsbiomaterials.2c01039 (PMC10015427; doi:10.1021/acsbiomaterials.2c01039)
Supplement: Supplementary file 1 — ab2c01039_si_001.pdf [file ab2c01039_si_001.pdf]

Supporting Information

# Biomimetic Dentin Repair: Amelogenin-Derived Peptide Guides Occlusion and Peritubular Mineralization of Human Teeth

Deniz T. Yucesoy*, Hanson Fong, John Hamann, Eric Hall, Sami Dogan & Mehmet Sarikaya*

**S1 – Peptide Design & Characterization:** The peptide sADP5, shortened ADP5, was designed using a procedure that was developed for deriving peptides from natural proteins, e.g., amelogenin, as described previously.[1] This approach enables  a knowledge-based design of short- peptides that are derived from natural proteins to capture their key functions.[1-2] Briefly, the first-generation peptides (7- & 12-AA) with affinity to HAp mineral, are combinatorially selected using phage- and cell-surface display directed evolution libraries. This is followed by designing the second-generation peptides with HAp biomineralizing characteristics using newly developed similarity matrices. Similarity matrix predicts the AA substitutions favorable towards binding. Unlike traditional matrices, e.g., PAM 250 and BLOSUM 62 which attempt to predict similarity based on the rate of AAs substitutions within proteins that have a similar function among species, in this method, the similarity matrix was constructed based on mutabilities of AAs using a "Markov chain model" without substantial overfitting typically caused by traditional matrices.[2] These matrices were used to systematically compare and identify similarity regions, i.e., domains of similar AA sequences between the HABPs and Amelogenin protein (rM180). The comparisons yielded high and low similarity regions along the amelogenin. By overlapping the high-similarity regions from both libraries, putative crystal binding sequences were identified and further refined by other computational tools, e.g., structure prediction, meta-functional signature, and ion binding domains analyses. As a result, a total of eight individual peptides were identified within the amelogenin, dubbed as Amelogenin-Derived Peptides (ADP's). It is noted here that, contrary to other peptide-based biomineralization approaches, which mainly utilizes random AAs and domains extracted from amelogenin with no systematic and iterative design

procedures (i.e., lack of quantitative binding and biomineralization data), in this approach the peptides that were identified through the combination of experimental and computational approaches which have been rigorously refined and characterized quantitatively to assess their predictive functions by both solid-binding and mineralization assays. While the set of ADPs derived from high similarity regions exerted high HAp binding affinity (first function of amelogenin), one particular peptide, ADP5, which was derived from low similarity region, has been found to have catalytic and fast kinetics characteristics in controlling HAp mineralization (second function of amelogenin). Table 1 (manuscript) shows the amino acid sequence and physical characteristics of sADP5 peptide. To further improve the solubility of ADP5 in aqueous solutions, an essential propensity for future clinical application, the six amino acids from the amino-end and alanine from the carboxyl-end were eliminated, while keeping the charged amino acids intact that are thought to initiate mineralization. Briefly, 0.8 µM peptide solution was mixed with an equal volume of mineralization solution to containing 48 mM CaCl2.2H2O and 28.8 mM β-Glycerophosphate (β-GP) in 25 mM Tris-HCl buffer (pH 7.4). The mineralization reaction was started by adding 0.10 U/µl bacterial Alkaline Phosphatase (AP, Invitrogen, USA) into 200 µl of reaction mixture. As the negative control, an equal volume of 25 mM Tris-HCl buffer (pH 7.4) was added onto mineralization solution. Recombinant amelogenin (rm180), original ADP5 and phage-display selected HABP1 with slow kinetics, were used as internal controls. 10 µl of the reaction solution was collected at 15-, 30-, 60- and 90-minutes. The reaction is stopped by quenching the AP activity by heating the solution to 90 °C and then rapidly cooling down to −20 °C. The mineral phase was removed by centrifugation and the unreacted ionic calcium in the supernatant was measured using QuantiChrome Calcium Assay Kit (Bioassays, USA).

**S2 – Peptide Synthesis and Purification:** Peptide synthesis was carried out on a preloaded Wang-resin using *Fmoc*/HBTU chemistry. The coupling efficiency was monitored by UV absorbance at 301 nm and the Fmoc deprotection was enabled using 20% (v/v) piperidine in DMF solution. The cleavage cocktail containing TFA/thioanisole/$H_2O$/phenol/ethanedithiol (87.5:5:5:2.5) was used for removal of resin that is followed by filtration. The crude peptide was then precipitated with cold ether and lyophilized for storage (Virtis Benchtop K, SP Industries, Inc., Warminster, PA). Purification was performed using a reverse-phase HPLC with a linear gradient of 1% increase per min at 10 mL/min flow rate. Retention times spanned 26-30 minutes depending on the peptide batch in semi-preparative HPLC. The mass verification is done by MALDI-TOF mass spectrometry with reflectron (RETOF-MS) on an Autoflex II (Bruker Daltonics, Billerica, MA) mass spectrometer in positive-ion mode. The observed fractions were subsequently collected manually from a scaled semi-preparative separation (Waters Deltaprep 600, semi-prep mode). The Supplementary Figure 1 demonstrates a representative MALDI Mass spectrum of sADP5 showing purified peptide peak at 1735.94 kDa (m/z).

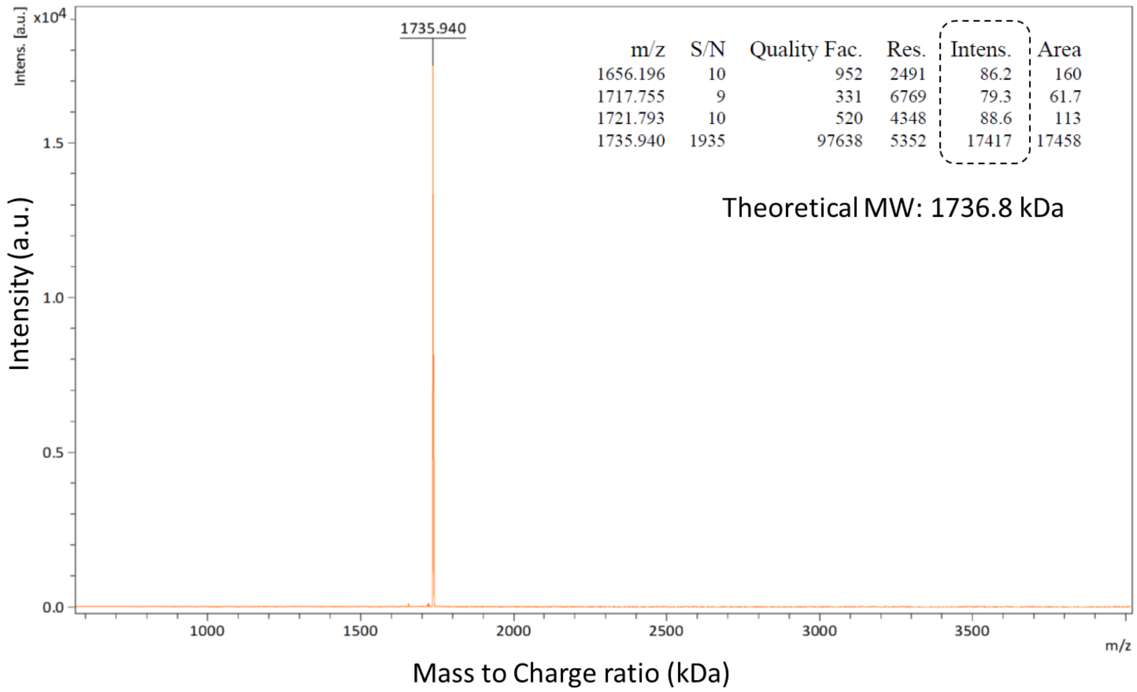


**Supplementary Figure 1:** Representative MALDI Mass spectrum of sADP5 showing purified peptide peak at 1735.94 kDa (m/z).

**S3 – Sample Preparation and Remineralization Procedure:**

Extracted human molar teeth (excluding third molars) with no visible defects or restorations were collected from University of Washington School of Dentistry Clinics within a disinfectant solution (10% (v/v) bleach) and stored in deionized water. Dentin discs with a thickness of ~2mm were cut using a low-speed saw (IsoMet™, Buehler, Lake Bluff, IL, USA) with a diamond blade from mid-coronal dentin. Coronal surface was then polished to 0.1 µm finish using diamond lapping films. Specimens were ultrasonicated for 2 minutes in order to remove the smear layer and then etched for 30 seconds with 10% (w/v) citric acid solution. Excess acid was removed by rinsing the samples with deionized water. For remineralization treatment, specimens were pre-wetted with 20mM Tris Buffer Solution, pH:7.4 (TBS) and then placed into 0.8 mM sADP5 peptide solution for 10 minutes at 37ºC. After removing the excess peptide by blotting, treatment samples were transferred into 20 mM TBS containing 3.22 mM $Ca^{2+}$ / 1.92 mM $PO_4^{3-}$ for 1 hour at 37°C. For repeated rounds of remineralization, samples were subjected to remineralization treatment immediately after water rinse. The mineralized samples were prepared in cross-section to simultaneously reveal both the dentin tubules and the newly formed mineral surface layer.

**S4 – EDXS Analysis of Geological Apatite:**

As a reference, freshly cleaved geological apatite was utilized for elemental analysis using energy-dispersive X-Ray spectroscopy (EDXS). The Supplementary Figure 2 shows Calcium to Phosphorus (Ca/P) ratio of 1.64 ± 0.08 as averaged from 3 data points which is in close agreement with the stoichiometric ratio of (Ca/P) in hydroxyapatite ($Ca_{10}(PO_4)_6(OH)_2$.

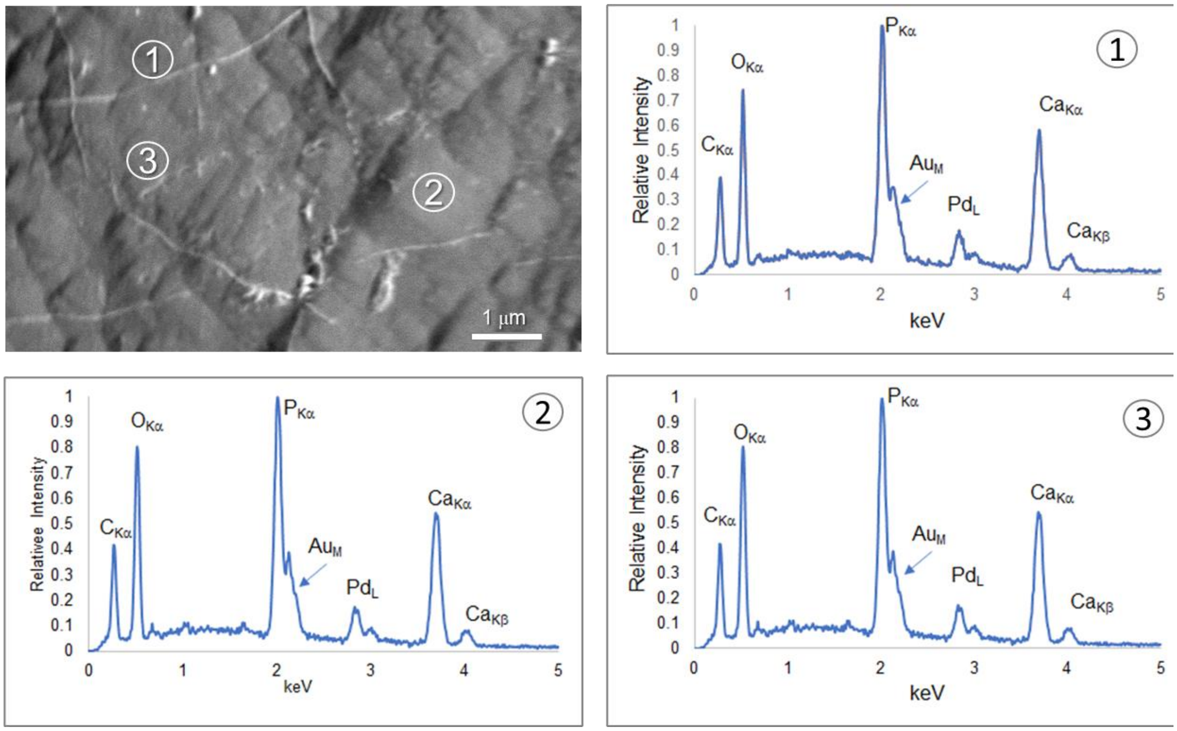


**Supplementary Figure 2:** EDXS Analysis: Elemental composition analysis of cleaved geological apatite with calcium to phosphorus ratio of Ca/P = 1.64 ± 0.08, averaged from 3 data points depicted as 1, 2, 3.

**S5 – Nanomechanical Characterization via Nanoindentation Testing:**

Nanoindentation testing provides a dynamic measurement with the indentation system tracking continuously load vs. depth during the indentation process, resulting in a force-depth (F-D) profile of the mechanical response of the sample, including biological hard tissues such as enamel, dentin, and bone. The values of hardness and elastic modulus were extracted from the linear extrapolation of the unloading curve.[4] Hardness obtained by this method contains partial elastic deformation as well as plastic deformation (Supplementary Figure 3). With small indentation depth and footprint, indentation characterization of the mineral layer was possible in cross-section.[3-4]

Additional relevant parameters used in this study were as follows:

1. Indentor tip was a Berkovich diamond tip with a tip radius at the apex = 90 nm

2. Hardness, *H,* and reduced elastic modulus, $E_r$, were calculated using the relationships reported by Oliver and Pharr[4]:

$$H = \frac{L}{A_c} \qquad (1)$$

$$E_r = \frac{S}{2}\left(\frac{\pi}{A_c}\right)^{1/2} \qquad (2)$$

Where *L* is the maximum applied load and *S* is the stiffness of the system. The area of contact, $A_c$, is calibrated as a function of contact depth, $h_c$**,** up to 200 nm.

Prior to measurements, the fractured remineralized dentin discs were mounted in a room temperature-cure epoxy, and the cross-section was polished to 0.1 μm finish using diamond lapping films (Allied High-Tech Products Inc., Rancho Dominguez, CA, USA). Nanoindentation measurements were made using a Triboindentor nanoindentation system (Hysitron Inc., Minneapolis, MN, USA) in air. The measurements were conducted on samples in cross-sectional geometry, i.e., indentor direction being parallel to the surface plane in spatially selected regions as the test facilitates scanned surface images. In order to volume

dependent measurements, maximum indentation depth for all measurements kept at 100 ± 20 nm. All reported H and $E_r$ values were averaged over 20 measurements.

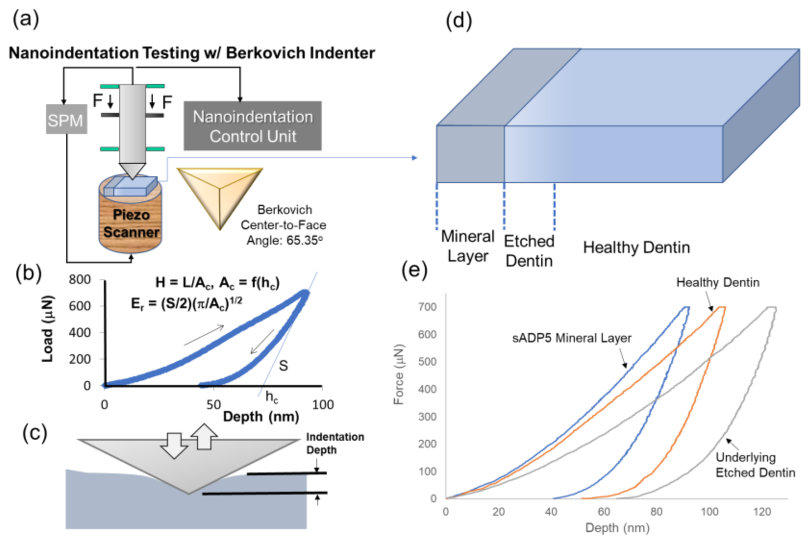


**Supplementary Figure 3:** Nanoindentation Procedure: (a) The nanoindentation apparatus, (b) Load-depth curve (F-D), and (c) A profile of an indentation. (d) Schematics of the samples characterized showing healthy (sound) dentin, demineralized dentin and newly formed mineral. (e) Examples of force-depth curves.

## S6 – Mineral Durability Characterization via Thermal Cycling

The durability of the mineralized layer formed on the exposed dentin was characterized through a thermal cycling assay that is adapted from ISO/TR dental materials testing procedure.[5] The test was designed to simulate the natural environment of the oral cavity by mimicking the physiological conditions when exposed to hot and cold cycling between the two extremes of 5°C and 55°C during daily exposure. Briefly, demineralized dentin discs obtained from human coronal dentin were subjected to three rounds of peptide-guided remineralization.

Following remineralization treatment, samples were fractured into two halves from the notch sides. Both halves were placed in artificial saliva (containing 130 mM KCl, 20 mM HEPES, 1.5 mM $CaCl_2$, 0.9 mM $KH_2PO_4$ and 1 mM NaCl; pH:7.0). While the control group sample was kept at 4°C, the second-half was subjected to thermal cycling. Aging protocol involves placement of the test tube containing the test specimen soaked in artificial saliva and cooling down and heating up the temperature to 5°C and 55°C for 200 and 2500 cycles which simulates 6 days and 3 months thermal stress in the oral environment. The test sample was dried under vacuum and fractured to assess the persistence/durability of the mineralized layer under thermal stresses developed during the thermal cycling process from the cross-sectional view.

**S7 – Thermal Strain and Stress Mismatch Analysis.**

A thin film approach is applied to estimate the thermal mismatch strain that results from the cyclic change in temperature. In absence of a direct measurement of the coefficient of thermal expansion (CTE) for the mineralized layer, a scaled CTE relative to that of enamel is used to estimate thermal mismatch strain and stress developed in the thin layers.[6] To inform the scaling of the CTE for the mineralized layer we exploit the relative hardness measured through nanoindentation to deduce the relative density of hydroxyapatite within the measured layer.[3,7] Assuming the density of hydroxyapatite within the mineralized layer plays a significant role in the thermal expansion properties we can develop a highly simplified approach to estimate the CTE using the CTE of enamel as a reference.[8] The true value for the CTE of the mineralized layer needs to be directly measured using a dynamic mechanical analysis instrument.

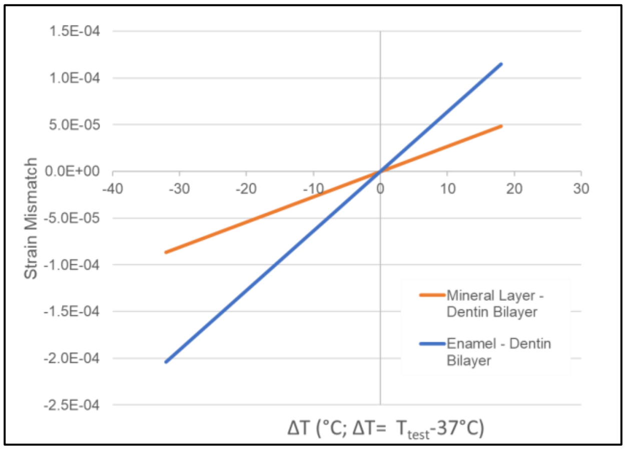


**Supplementary Figure 4.** Comparison of the estimated thermal mismatch strain of the mineralized layer and dentin with that of enamel and dentin. The estimated thermal mismatch strain is presented across the range of temperatures assuming the mineralized layer formed at 37°C and no lattice mismatch strain was developed as a result of the growth.

$$\alpha_{min} = \frac{H_{min}}{H_{enamel}} * \alpha_{enamel} \qquad (3)$$

To illustrate the effect of thermal expansion we consider that mineralization of the new layer on dentin occurred at 37°C and developed no initial strain due to lattice mismatch. Thermal cycling was conducted between 5 to 55°C so we can translate this range to that of temperature change relative to mineralization by shifting down 37°C to arrive at a temperature change range of -32 to 18°C. In Supplementary Figure 4, we compare the estimated thermal mismatch strain of the mineralized layer and dentin with that of enamel and dentin. Figure 6b (see main text) shows an estimation of the individual strain that developed each respective material. In Figure 6c (see main text) we compare the estimated stress developed in the mineralized layer

and dentin resulting from the thermal mismatch strain. Note that we exploited the reduced elastic modulus only to illustrate the effect of relative stiffness between the mineralized layer and dentin. However, this is not representative of the real stress developed in the system. Note: mean values for parameters were used throughout.

Assumptions:

- 1D thin film model
- Measured CTE is analogous to linear CTE
- Each material is isotropic
- The interface is coherent
- The CTE of the mineralized layer can be approximated by scaling the CTE of enamel by the relative hardness of the mineralized layer to enamel

This analysis was contrived to estimate the magnitude of the strain and stress that may be experienced by the material. The thermal mismatch strain with dentin and the mineralized layer is estimated to be less than that of enamel over the same range of temperature. This provides an estimated range of thermal strain of 0.0002 in tension to 0.0004 in compression for the mineralized layer. Considering the relative stiffness of the materials yields no further insight apart from providing an estimated range of stresses between 2 MPa in tension to 3 MPa in compression for the mineralized layer.

This is an obvious conclusion to draw given we are only comparing the scaled CTE to the unscaled CTE of enamel. However, if we consider the increased porosity of the mineralized layer as evidenced by the SEM imagery, we can justify our scaled CTE by considering that increased space between hydroxyapatite crystallites leads to a higher degree of strain localization compared to that of the more crystalline enamel. We believe strain delocalization plays a significant role in the reduction of thermal mismatch strain across the mineralized interface which contributes to the structural integrity observed over thermal cycling. Intercalation of the mineralized layer with the dentin tubules is likely to further increase structural integrity but was not considered as a factor in this analysis.

**References:**

(1) Gungormus, M.; Oren, E. E.; Horst, J. A.; Fong, H.; Hnilova, M.; Somerman, M. J.; Snead, M. L.; Samudrala, R.; Tamerler, C.; Sarikaya, M., Cementomimetics—constructing a cementum-like biomineralized microlayer via amelogenin-derived peptides. *Int J Oral Sci.* **2012,** *4* (2), 69-77, DOI: 10.1038/ijos.2012.40.

(2) Oren E. E.; Tamerler C.; Sahin D.; Hnilova M.; Seker U. O. S.; Sarikaya M., Samudrala R. A novel knowledge-based approach to design inorganic-binding peptides. *Bioinformatics* **2007** *23* (21), 2816-2822, DOI: 10.1093/bioinformatics/btm436.

(3) Fong, H.; Sarikaya, M.; White, S. N.; Snead, M. L., Nano-mechanical properties profiles across dentin–enamel junction of human incisor teeth. *Mater Sci Eng C.* **1999,** *7* (2), 119-128, DOI: 10.1016/S0928-4931(99)00133-2.

(4) Oliver W. C.; Pharr G. M., An improved technique for determining hardness and elastic modulus using load and displacement sensing indentation experiments. *J Mater Res* **1992** *7*(6), 1564-1583, DOI: 10.1557/JMR.1992.1564.

(5) International Standard Organization ISO/TS 11405. Dentistry – testing of adhesion to tooth structure. Geneve, Switzerland, **2015.**

(6) Freund L. B.; Suresh S., Thin film materials: Stress, defect formation and surface evolution. Cambridge University Press, **2004,** DOI: 10.1017/CBO9780511754715.

(7) He, L. H.; Fujisawa, N.; Swain, M. V., Elastic modulus and stress–strain response of human enamel by nano-indentation. *Biomaterials* **2006,** *27* (24), 4388-4398, DOI: 10.1016/j.biomaterials.2006.03.045.

(8)Hengchang X.; Wenyi L.; Tong W., Measurement of thermal expansion coefficient of human teeth. *Aust Dent J.* **1989** 34 (6):530-535, DOI: 10.1111/j.1834-7819.1989.tb04660.x.
